# Supplementary material for: Socioeconomic position and adverse childhood experiences as risk factors for health-related behaviour change and employment adversity during the COVID-19 pandemic: insights from a prospective cohort study in the UK
Source: BMC Public Health. 2022 Sep 24;22:1820. doi: 10.1186/s12889-022-14184-8 (PMC9509623; doi:10.1186/s12889-022-14184-8)
Supplement: Supplementary file 1 — Additional file 1: Supplementary Fig. 1. Flow chart of inclusion and exclusion of participants. Supplementary Table 1. Recoding of 8-class NS-SEC variables to 4 classes as defined by UK Office for National Statistics. Supplementary Table 2. Participant characteristics. Characteristics of the participants included in SEP analyses using data from multi-variate multiple imputation. N = 2557. Supplementary Table 3. Characteristics of participants that responded to COVID questionnaire 2, before imputation. N = 2710. Supplementary Table 4. Maternal education for excluded participants and participants included in analyses. Supplementary Table 5. Association between social class and changes in health-related behaviour during the March–July 2020 lockdown (adjusted for ethnicity, age at time of questionnaire, home ownership, maternal and partner education, parity, maternal age and maternal marital status). N = 2557. Supplementary Table 6. Association between Adverse Childhood Experiences score and changes in health-related behaviour during the March–July 2020 lockdown (adjusted for ethnicity, age at time of questionnaire, home ownership, maternal and partner education, parity, maternal age and maternal marital status). N = 2707. Supplementary Table 7. Association between individual Adverse Childhood Experiences and changes in health-related behaviour during the March–July 2020 lockdown (unadjusted). N = 2707. Supplementary Table 8. Association between individual Adverse Childhood Experiences and changes in health-related behaviour during the March–July 2020 lockdown (adjusted for ethnicity, age at time of questionnaire, home ownership, maternal and partner education, parity, maternal age and maternal marital status). N = 2707. Supplementary Table 9. Association between social class and changes in financial situation during the March–July 2020 lockdown (adjusted for ethnicity, age at time of questionnaire, home ownership, maternal and partner education, parity, maternal age an [file 12889_2022_14184_MOESM1_ESM.pdf]

Supplmentary figures and tables

Socioeconomic position and adverse childhood experiences as risk factors for health-related behavior change and employment adversity during the COVID-19 pandemic: insights from a prospective cohort study in the UK

Smith et al.

Supplementary Figure 1: Flow chart of inclusion and exclusion of participants

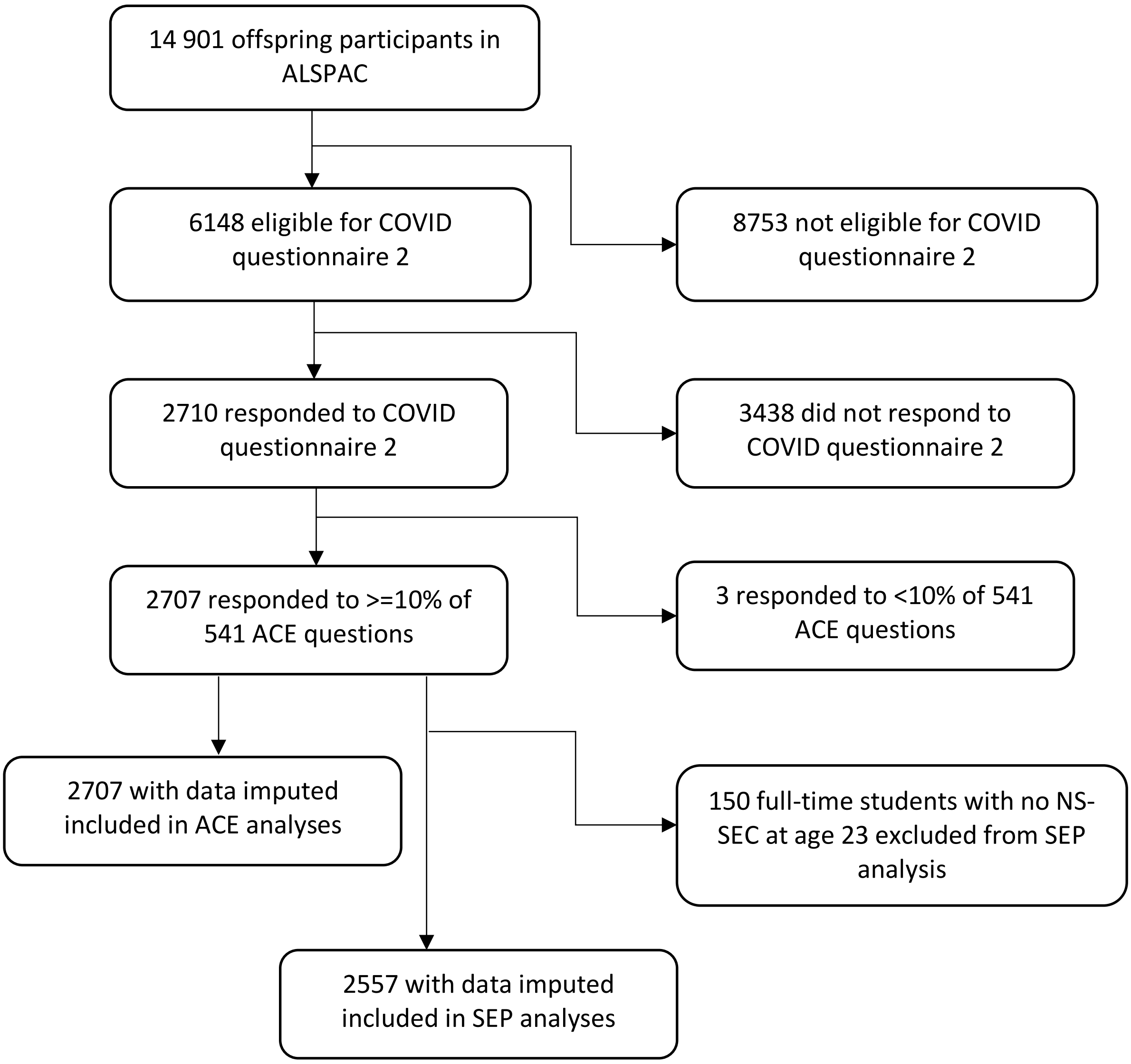

Supplementary Table 1. Recoding of 8-class NS-SEC variable to 4 classes as defined by the UK Office for National Statistics.

8 Class NS-SEC		3 Class NS-SEC	
**Category**	**n (%)**	**Category**	**n (%)**
1. Higher managerial, administrative and professional occupations	313 (12.2)	1. Higher managerial, administrative and professional occupations	1126 (44.0)
2. Lower managerial, administrative and professional occupations	817 (32.0)		
3. Intermediate occupations	586 (22.9)	2. Intermediate occupations	616 (24.1)
4. Small employers and own account workers	30 (1.2)		
5. Lower supervisory and technical occupations	94 (3.7)	3. Routine and manual occupations	579 (22.6)
6. Semi-routine occupations	321 (12.6)		
7. Routine occupations	160 (6.3)		
8. Never worked and long-term unemployed	236 (9.2)	Never worked and long-term unemployed	236 (9.2)

Supplementary Table 2. Characteristics of participants included in SEP analyses using data from multi-variate imputation. N=2557

Variable	n (%)				
**Sex**	**Female**	**Male**			
	1815 (71.0)	742 (29.0)			
**Age (years)**	**27**	**28**	**29**		
	827 (32.3)	1479 (57.8)	251 (9.8)		
**Ethnicity**	**White**	**Non-white**			
	2450 (95.8)	107 (4.2)			
**Socio-economic status (NS-SEC)**	**1. Higher managerial, administrative and professional occupations**	**2. Intermediate occupations**	**3. Routine and manual occupations**	**Never worked and long-term unemployed**	
	1126 (44.0)	616 (24.1)	579 (22.6)	236 (9.2)	
**Health-related behaviours**	**Decreased**	**Stayed the same**	**Increased**		
Number of homecooked meals	92 (3.4)	860 (31.8)	1605 (59.3)		
Number of meals	262 (9.7)	1827 (67.5)	468 (17.3)		
Number of snacks	347 (12.8)	948 (35.0)	1262 (46.6)		
Exercise quantity	1030 (38.0)	491 (18.1)	1036 (38.3)		
Sleep quantity	608 (22.5)	1073 (39.6)	876 (32.4)		
	**Decreased**	**Stayed the same**	**Increased**	**Non-drinker/non-smoker**	
Alcohol quantity	428 (15.8)	789 (29.1)	987 (36.5)	353 (13.0)	
Smoking/vaping quantity	99 (3.7)	273 (10.1)	289 (10.7)	1896 (70.0)	
**Financial/employment variables**					
Change in employment status	**Employed no change**	**Employed reduced hours**	**Furlough/paid leave/unpaid leave**	**Stopped working during pandemic**	**Not working pre-pandemic**
	1473 (54.4)	252 (9.3)	481 (17.8)	166 (6.1)	185 (6.8)
Financial situation since pandemic	**Worse off**	**Stayed the same**	**Better off**		
	664 (24.5)	954 (35.2)	939 (4.7)		
Claimed benefits since pandemic	340 (12.6)				
Mortgage/rent deferral since pandemic	178 (6.6)				

Supplementary Table 3. Characteristics of participants that responded to COVID questionnaire 2, before imputation. N=2710. *Continued on next page.*

**Variable**	**n (%)**								
**Sex**	**Female**	**Male**							**Missing**
	1912 (70.6)	795 (29.3)							3 (0.1)
**Age (years)**	**27**	**28**	**29**						**Missing**
	888 (32.8)	1557 (57.5)	265 (9.8)						0 (0.0)
**Ethnicity**	**White**	**Non-white**							**Missing**
	2326 (85.8)	81 (3.0)							303 (11.2)
**Obtained GCSE grades A*-C**[a]	**Yes**	**No**							**Missing**
	1596 (58.9)	38 (1.4)							1076 (39.7)
**Monthly take-home pay (£)**[a]	**Not doing paid work**	**1-499**	**500-999**	**1000-1499**	**1500-1999**	**2000-2499**	**2500-2999**	**3000+**	**Missing**
	49 (1.8)	102 (3.8)	205 (7.6)	693 (25.6)	665 (24.5)	210 (7.7)	69 (2.5)	41 (1.5)	676 (24.9)
**ACE score**	**0**	**1**	**2-3**	**4+**					**Missing**
	341 (12.6)	407 (15.0)	517 (19.1)	192 (7.1)					1253 (46.2)
**ACEs**									**Missing**
Physical abuse	401 (14.8)								649 (23.9)
Sexual abuse	111 (4.1)								349 (12.9)
Emotional abuse	411 (15.2)								649 (23.9)
Emotional neglect	368 (13.6)								680 (25.1)
Bullying	506 (18.7)								540 (19.9)
Violence between parents	335 (12.4)								811 (29.9)
Parental substance abuse	167 (6.3)								662 (24.4)
Parental mental illness or suicide	873 (32.2)								606 (22.4)
Parental criminal conviction	138 (5.1)								616 (22.7)
Parental separation	469 (17.3)								758 (28.0)
**Socio-economic status (NS-SEC)**	**1. Higher managerial, administrative and professional occupations**	**2. Intermediate occupations**	**3. Routine and manual occupations**	**Never worked and long-term unemployed**					**Missing**
	855 (31.5)	471 (17.4)	437 (16.1)	163 (6.0)					784 (28.9)
**Health-related behaviours**	**Decreased**	**Stayed the same**	**Increased**						**Missing**

<table>
<tr><td>Number of homecooked meals</td><td colspan="2">96 (3.5)</td><td colspan="5">885 (32.7)</td><td colspan="2">1654 (61.0)</td><td>75 (2.8)</td></tr>
<tr><td>Number of meals</td><td colspan="2">264 (9.7)</td><td colspan="5">1904 (70.3)</td><td colspan="2">469 (17.3)</td><td>73 (2.7)</td></tr>
<tr><td>Number of snacks</td><td colspan="2">346 (12.8)</td><td colspan="5">992 (36.6)</td><td colspan="2">1293 (47.7)</td><td>79 (2.9)</td></tr>
<tr><td>Exercise quantity</td><td colspan="2">1058 (39.0)</td><td colspan="5">508 (18.7)</td><td colspan="2">1071 (39.5)</td><td>73 (2.7)</td></tr>
<tr><td>Sleep quantity</td><td colspan="2">910 (33.6)</td><td colspan="5">1112 (41.0)</td><td colspan="2">910 (33.6)</td><td>76 (2.8)</td></tr>
<tr><td></td><td>Decreased</td><td colspan="3">Stayed the same</td><td colspan="3">Increased</td><td colspan="2">Non-drinker/non-smoker</td><td>Missing</td></tr>
<tr><td>Alcohol quantity</td><td>449 (16.6)</td><td colspan="3">812 (30.0)</td><td colspan="3">1021 (37.7)</td><td colspan="2">357 (13.2)</td><td>71 (2.6)</td></tr>
<tr><td>Smoking/vaping quantity</td><td>101 (3.7)</td><td colspan="3">279 (10.3)</td><td colspan="3">286 (10.6)</td><td colspan="2">1971 (72.7)</td><td>73 (2.7)</td></tr>
<tr><td colspan="10">Financial/employment variables</td><td></td></tr>
<tr><td rowspan="2">Change in employment status</td><td>Employed no change</td><td colspan="2">Employed reduced hours</td><td colspan="3">Furlough/paid leave/unpaid leave</td><td colspan="2">Stopped working during pandemic</td><td>Not working pre-pandemic</td><td>Missing</td></tr>
<tr><td>1506 (55.6)</td><td colspan="2">247 (9.1)</td><td colspan="3">460 (17.0)</td><td colspan="2">158 (5.8)</td><td>184 (6.8)</td><td>155 (5.7)6</td></tr>
<tr><td rowspan="2">Financial situation since pandemic</td><td colspan="2">Worse off</td><td colspan="5">Stayed the same</td><td colspan="2">Better off</td><td>Missing</td></tr>
<tr><td colspan="2">639 (23.6)</td><td colspan="5">968 (35.7)</td><td colspan="2">969 (35.8)</td><td>134 (4.9)</td></tr>
<tr><td rowspan="2">Claimed benefits since pandemic</td><td colspan="5">Yes</td><td colspan="4">No</td><td>Missing</td></tr>
<tr><td colspan="5">306 (11.3)</td><td colspan="4">2250 (83.0)</td><td>154 (5.7)</td></tr>
<tr><td rowspan="2">Mortgage/rent deferral since pandemic</td><td colspan="5">Yes</td><td colspan="4">No</td><td>Missing</td></tr>
<tr><td colspan="5">167 (6.2)</td><td colspan="4">2413 (89.0)</td><td>130 (4.8)</td></tr>
</table>

[a]Details on the data collection for these additional demographic variables are in Additional File 2.

Supplementary Table 4. Maternal education for excluded participants and participants included in analyses.

	Participants (%)	
	**Excluded**	**Included**
	N=12911	N=2710
**Mother's highest education qualification**		
CSE	13.6	8.2
Vocational	7.0	6.2
O level	24.3	30.3
A level	14.9	25.1
Degree	7.6	20.1
Not available	32.6	10.1

Supplementary Table 5: Association between social class and changes in health-related behaviour during the March-July 2020 lockdown (adjusted for ethnicity, age at time of questionnaire, home ownership, maternal and partner education, parity, maternal age and maternal marital status). N=2557

	**NS-SEC social class at age 23 years**			
	**Higher managerial, administrative and professional occupations**	**Intermediate occupations**	**Routine and manual occupations**	**Never worked and long-term unemployed**
	**N=1126**	**N=616**	**N=579**	**N=236**
**Number of home-cooked meals**				
Decreased	ref	0.79(0.42-1.47)	0.63(0.31-1.28)	0.58(0.21-1.57)
Stayed the same	ref	ref	ref	ref
Increased	ref	0.97(0.76-1.25)	0.77(0.59-1.00)	0.72(0.51-1.04)
**Number of meals**				
Decreased	ref	1.03(0.69-1.54)	1.41(0.95-2.09)	1.22(0.67-2.23)
Stayed the same	ref	ref	ref	ref
Increased	ref	0.91(0.67-1.24)	1.13(0.83-1.55)	1.26(0.81-1.97)
**Number of snacks**				
Decreased	ref	1.23(0.86-1.76)	1.09(0.73-1.62)	0.85(0.45-1.59)
Stayed the same	ref	ref	ref	ref
Increased	ref	1.02(0.80-1.31)	1.02(0.80-1.31)	0.99(0.69-1.43)
**Exercise quantity**				
Decreased	ref	1.08(0.77-1.50)	1.02(0.73-1.42)	0.83(0.52-1.33)
Stayed the same	ref	ref	ref	ref
Increased	ref	1.11(0.81-1.52)	0.99(0.72-1.37)	0.78(0.50-1.23)
**Sleep quantity**				
Decreased	ref	1.16(0.86-1.55)	1.26(0.93-1.70)	1.41(0.92-2.16)
Stayed the same	ref	ref	ref	ref
Increased	ref	1.12(0.87-1.44)	0.99(0.75-1.30)	1.11(0.73-1.67)
**Alcohol quantity**				
Decreased	ref	1.09(0.78-1.52)	1.00(0.68-1.47)	1.02(0.61-1.72)
Stayed the same	ref	ref	ref	ref
Increased	ref	1.08(0.82-1.42)	1.08(0.82-1.42)	0.74(0.48-1.16)
Non-drinker	ref	1.08(0.74-1.59)	1.36(0.92-2.02)	1.42(0.83-2.44)
**Smoking/vaping quantity**				
Decreased	ref	1.39(0.68-2.84)	1.11(0.53-2.35)	1.68(0.7-4.04)
Stayed the same	ref	ref	ref	ref
Increased	ref	0.97(0.57-1.65)	0.86(0.53-1.41)	0.93(0.47-1.82)
Non-smoker	ref	0.89(0.59-1.34)	0.58(0.40-0.86)	0.48(0.29-0.79)

Supplementary Table 6: Association between Adverse Childhood Experiences score and changes in health-related behaviour during the March-July 2020 lockdown (adjusted for ethnicity, age at time of questionnaire, home ownership, maternal and partner education, parity, maternal age and maternal marital status). N=2707

	**Number of Adverse Childhood Experiences between 0-16 years**			
	**0**	**1**	**2-3**	**4 or more**
	**N=504**	**N=679**	**N=980**	**N=544**
**Number of home-cooked meals**				
Decreased	ref	1.15(0.55-2.41)	1.25(0.63-2.50)	1.44(0.64-3.24)
Stayed the same	ref	ref	ref	ref
Increased	ref	0.99(0.74-1.31)	1.16(0.89-1.51)	0.87(0.64-1.19)
**Number of meals**				
Decreased	ref	1.21(0.72-2.04)	1.59(1.01-2.51)	1.90(1.12-3.23)
Stayed the same	ref	ref	ref	ref
Increased	ref	1.34(0.92-1.95)	1.35(0.95-1.91)	1.61(1.07-2.41)
**Number of snacks**				
Decreased	ref	1.10(0.73-1.64)	1.17(0.80-1.72)	1.21(0.76-1.91)
Stayed the same	ref	ref	ref	ref
Increased	ref	1.02(0.77-1.36)	1.08(0.84-1.40)	1.06(0.76-1.47)
**Exercise quantity**				
Decreased	ref	1.02(0.71-1.48)	0.99(0.70-1.40)	1.08(0.71-1.65)
Stayed the same	ref	ref	ref	ref
Increased	ref	0.96(0.67-1.39)	0.98(0.71-1.36)	1.06(0.70-1.63)
**Sleep quantity**				
Decreased	ref	1.00(0.70-1.43)	1.08(0.77-1.50)	1.39(0.94-2.06)
Stayed the same	ref	ref	ref	ref
Increased	ref	0.97(0.72-1.32)	1.06(0.80-1.41)	1.06(0.75-1.52)
**Alcohol quantity**				
Decreased	ref	0.98(0.66-1.45)	0.90(0.62-1.30)	1.39(0.89-2.16)
Stayed the same	ref	ref	ref	ref
Increased	ref	0.98(0.71-1.34)	1.08(0.80-1.46)	1.34(0.92-1.96)
Non-drinker	ref	0.81(0.52-1.24)	0.88(0.59-1.32)	1.55(0.95-2.53)
**Smoking/vaping quantity**				
Decreased	ref	1.05(0.45-2.48)	0.99(0.47-2.10)	1.00(0.41-2.45)
Stayed the same	ref	ref	ref	ref
Increased	ref	1.35(0.68-2.68)	1.18(0.65-2.14)	1.82(0.94-3.51)
Non-smoker	ref	0.99(0.61-1.60)	0.67(0.44-1.00)	0.63(0.38-1.04)

Supplementary Table 7: Association between individual Adverse Childhood Experiences and changes in health-related behaviour during the March-July 2020 lockdown (unadjusted). N=2707

	**Adverse Childhood Experience**									
	**Physical abuse**	**Sexual abuse**	**Emotional abuse**	**Emotional neglect**	**Bullying**	**Violence between parents**	**Parental substance abuse**	**Parental mental illness or suicide**	**Parental criminal conviction**	**Parental separation**
	**N=622**	**N=163**	**N=659**	**N=529**	**N=646**	**N=604**	**N=330**	**N=1183**	**N=280**	**N=780**
**Number of home-cooked meals**										
Decreased	1.12(0.63-1.98)	1.45(0.61-3.45)	1.22(0.71-2.08)	1.61(0.93-2.77)	1.31(0.80-2.16)	1.57(0.91-2.71)	1.23(0.57-2.66)	1.03(0.65-1.63)	1.36(0.62-2.99)	0.70(0.40-1.24)
Stayed the same	ref	ref	ref	ref	ref	ref	ref	ref	ref	ref
Increased	0.97(0.78-1.20)	0.95(0.63-1.42)	0.93(0.75-1.14)	0.76(0.60-0.98)	0.95(0.77-1.17)	0.95(0.75-1.19)	1.09(0.80-1.47)	0.97(0.81-1.15)	0.96(0.68-1.36)	0.84(0.68-1.03)
**Number of meals**										
Decreased	1.57(1.12-2.21)	1.69(0.97-2.92)	1.13(0.79-1.63)	1.64(1.17-2.31)	1.44(1.05-1.97)	1.42(0.99-2.05)	1.47(0.95-2.30)	1.41(1.05-1.89)	1.40(0.85-2.3)	1.24(0.89-1.73)
Stayed the same	ref	ref	ref	ref	ref	ref	ref	ref	ref	ref
Increased	1.37(1.05-1.81)	1.43(0.91-2.25)	1.43(1.11-1.84)	1.18(0.88-1.58)	1.31(1.02-1.68)	1.47(1.10-1.95)	1.21(0.84-1.76)	1.14(0.91-1.43)	0.96(0.61-1.51)	1.31(1.00-1.71)
**Number of snacks**										
Decreased	0.98(0.71-1.34)	1.48(0.88-2.50)	1.03(0.73-1.44)	1.01(0.71-1.44)	0.96(0.70-1.32)	1.10(0.76-1.58)	1.57(1.02-2.42)	1.19(0.90-1.56)	1.51(0.95-2.39)	0.98(0.70-1.36)

Stayed the same	ref	ref	ref	ref	ref	ref	ref	ref	ref	ref
Increased	0.94(0.74-1.20)	1.04(0.70-1.54)	1.01(0.81-1.26)	1.02(0.81-1.29)	1.11(0.90-1.38)	1.22(0.96-1.56)	1.29(0.95-1.74)	1.11(0.91-1.34)	0.96(0.66-1.40)	1.27(1.02-1.58)
**Exercise quantity**										
Decreased	0.91(0.69-1.22)	1.17(0.68-2.01)	1.16(0.88-1.54)	1.02(0.76-1.36)	1.07(0.80-1.41)	1.35(0.98-1.86)	1.30(0.86-1.96)	0.96(0.76-1.22)	0.80(0.51-1.25)	0.98(0.73-1.30)
Stayed the same	ref	ref	ref	ref	ref	ref	ref	ref	ref	ref
Increased	0.82(0.62-1.09)	1.28(0.76-2.16)	1.05(0.79-1.39)	0.76(0.56-1.04)	0.98(0.74-1.30)	1.28(0.93-1.76)	1.25(0.84-1.85)	1.09(0.86-1.37)	1.11(0.73-1.68)	0.89(0.68-1.18)
**Sleep quantity**										
Decreased	1.38(1.07-1.78)	1.48(0.94-2.32)	1.44(1.09-1.90)	1.00(0.77-1.32)	1.24(0.96-1.59)	1.38(1.04-1.84)	1.19(0.79-1.78)	1.24(0.99-1.54)	1.16(0.77-1.75)	1.03(0.79-1.35)
Stayed the same	ref	ref	ref	ref	ref	ref	ref	ref	ref	ref
Increased	1.03(0.81-1.32)	1.30(0.84-2.02)	1.12(0.88-1.41)	0.83(0.64-1.06)	0.96(0.76-1.20)	1.08(0.83-1.39)	1.18(0.86-1.62)	1.07(0.88-1.30)	1.06(0.72-1.55)	0.79(0.63-1.00)
**Alcohol quantity**										
Decreased	1.41(1.02-1.95)	0.99(0.56-1.75)	1.08(0.79-1.49)	1.11(0.79-1.57)	0.95(0.71-1.27)	1.65(1.19-2.28)	1.36(0.89-2.09)	0.96(0.74-1.23)	1.18(0.74-1.90)	0.96(0.70-1.31)
Stayed the same	ref	ref	ref	ref	ref	ref	ref	ref	ref	ref
Increased	1.37(1.06-1.78)	0.99(0.63-1.55)	1.22(0.95-1.58)	1.00(0.76-1.31)	0.87(0.68-1.10)	1.36(1.04-1.80)	1.26(0.88-1.81)	1.02(0.83-1.26)	0.97(0.65-1.44)	0.99(0.79-1.26)
Non-drinker	2.13(1.53-2.97)	1.71(1.02-2.88)	1.70(1.21-2.40)	1.59(1.10-2.30)	1.03(0.75-1.43)	1.41(0.96-2.06)	1.32(0.81-2.16)	0.97(0.72-1.31)	1.11(0.65-1.89)	1.37(0.98-1.90)

**Smoking/vaping quantity**										
Decreased	0.50(0.25-1.01)	0.50(0.13-1.86)	0.81(0.44-1.48)	1.23(0.69-2.21)	1.40(0.83-2.35)	1.35(0.72-2.54)	1.00(0.49-2.01)	0.98(0.59-1.61)	0.84(0.38-1.90)	0.72(0.40-1.30)
Stayed the same	ref	ref	ref	ref	ref	ref	ref	ref	ref	ref
Increased	1.10(0.72-1.68)	1.05(0.52-2.12)	1.17(0.78-1.76)	0.97(0.62-1.53)	1.16(0.78-1.74)	1.97(1.25-3.11)	1.36(0.82-2.26)	1.15(0.78-1.69)	0.85(0.45-1.59)	1.28(0.85-1.92)
Non-smoker	0.66(0.48-0.91)	0.72(0.42-1.25)	0.76(0.54-1.06)	0.75(0.53-1.06)	0.79(0.58-1.09)	1.04(0.72-1.51)	0.67(0.44-1.03)	0.81(0.60-1.09)	0.58(0.36-0.91)	0.65(0.47-0.90)

Supplementary Table 8: Association between individual Adverse Childhood Experiences and changes in health-related behaviour during the March-July 2020 lockdown (adjusted for ethnicity, age at time of questionnaire, home ownership, maternal and partner education, parity, maternal age and maternal marital status). N=2707

	**Adverse Childhood Experience**									
	**Physical abuse**	**Sexual abuse**	**Emotional abuse**	**Emotional neglect**	**Bullying**	**Violence between parents**	**Parental substance abuse**	**Parental mental illness or suicide**	**Parental criminal conviction**	**Parental separation**
	**N=622**	**N=163**	**N=659**	**N=529**	**N=646**	**N=604**	**N=330**	**N=1183**	**N=280**	**N=780**
**Number of home-cooked meals**										
Decreased	1.07(0.58-1.96)	1.39(0.56-3.43)	1.16(0.66-2.05)	1.63(0.91-2.92)	1.25(0.74-2.10)	1.66(0.93-2.98)	1.42(0.59-3.42)	1.01(0.62-1.63)	1.33(0.58-3.07)	0.72(0.38-1.34)
Stayed the same	ref	ref	ref	ref	ref	ref	ref	ref	ref	ref
Increased	1.00(0.79-1.25)	0.96(0.63-1.47)	0.94(0.75-1.17)	0.81(0.63-1.05)	0.95(0.76-1.18)	0.99(0.78-1.26)	1.17(0.83-1.64)	1.01(0.84-1.22)	1.00(0.69-1.44)	0.92(0.73-1.16)
**Number of meals**										
Decreased	1.43(1.00-2.04)	1.46(0.82-2.58)	0.98(0.67-1.45)	1.57(1.09-2.27)	1.40(1.01-1.95)	1.27(0.86-1.87)	1.16(0.69-1.95)	1.29(0.95-1.75)	1.22(0.72-2.08)	1.02(0.70-1.47)
Stayed the same	ref	ref	ref	ref	ref	ref	ref	ref	ref	ref
Increased	1.23(0.92-1.64)	1.26(0.78-2.05)	1.27(0.97-1.67)	1.05(0.77-1.44)	1.27(0.97-1.66)	1.29(0.94-1.75)	0.86(0.57-1.31)	1.01(0.80-1.28)	0.77(0.47-1.25)	1.01(0.75-1.37)
**Number of snacks**										

Decreased	0.93(0.67-1.29)	1.48(0.85-2.56)	0.99(0.69-1.41)	1.02(0.70-1.48)	0.95(0.68-1.31)	1.06(0.72-1.57)	1.47(0.90-2.39)	1.20(0.90-1.59)	1.46(0.89-2.38)	0.97(0.68-1.40)
Stayed the same	ref	ref	ref	ref	ref	ref	ref	ref	ref	ref
Increased	0.88(0.69-1.12)	0.95(0.63-1.44)	0.94(0.75-1.20)	0.96(0.75-1.23)	1.10(0.88-1.38)	1.13(0.87-1.46)	1.15(0.83-1.59)	1.06(0.87-1.29)	0.88(0.59-1.31)	1.14(0.90-1.45)
**Exercise quantity**										
Decreased	0.90(0.66-1.22)	1.06(0.60-1.88)	1.14(0.84-1.54)	1.01(0.74-1.37)	1.07(0.80-1.43)	1.41(1.00-2.00)	1.36(0.85-2.17)	0.94(0.73-1.21)	0.75(0.46-1.21)	0.97(0.70-1.33)
Stayed the same	ref	ref	ref	ref	ref	ref	ref	ref	ref	ref
Increased	0.83(0.62-1.12)	1.28(0.75-2.21)	1.06(0.79-1.42)	0.76(0.55-1.04)	0.97(0.73-1.30)	1.40(1.00-1.97)	1.43(0.91-2.23)	1.11(0.87-1.42)	1.15(0.73-1.79)	0.96(0.71-1.30)
**Sleep quantity**										
Decreased	1.28(0.99-1.67)	1.33(0.83-2.12)	1.39(1.05-1.86)	0.95(0.71-1.27)	1.21(0.94-1.56)	1.35(0.99-1.83)	1.09(0.68-1.75)	1.16(0.92-1.46)	1.05(0.67-1.63)	0.94(0.71-1.26)
Stayed the same	ref	ref	ref	ref	ref	ref	ref	ref	ref	ref
Increased	1.07(0.83-1.38)	1.40(0.89-2.22)	1.17(0.91-1.50)	0.83(0.64-1.08)	0.95(0.75-1.20)	1.12(0.85-1.47)	1.24(0.87-1.77)	1.12(0.92-1.38)	1.07(0.72-1.59)	0.84(0.65-1.09)
**Alcohol quantity**										
Decreased	1.42(1.02-1.98)	0.96(0.53-1.73)	1.04(0.75-1.46)	1.13(0.79-1.62)	0.92(0.68-1.25)	1.73(1.22-2.44)	1.34(0.83-2.18)	0.96(0.74-1.25)	1.15(0.70-1.88)	1.05(0.74-1.47)
Stayed the same	ref	ref	ref	ref	ref	ref	ref	ref	ref	ref
Increased	1.40(1.07-1.84)	1.01(0.63-1.62)	1.29(0.99-1.68)	1.01(0.76-1.33)	0.87(0.68-1.11)	1.39(1.04-1.87)	1.36(0.92-2.02)	1.02(0.82-1.26)	0.99(0.65-1.50)	1.01(0.78-1.32)

Non-drinker	1.95(1.38-2.77)	1.48(0.85-2.57)	1.59(1.10-2.3)	1.38(0.94-2.03)	0.98(0.70-1.37)	1.28(0.85-1.93)	1.06(0.60-1.89)	0.84(0.61-1.14)	0.96(0.53-1.71)	1.20(0.83-1.73)
**Smoking/vaping quantity**										
Decreased	0.52(0.25-1.09)	0.52(0.14-1.97)	0.77(0.40-1.47)	1.43(0.76-2.67)	1.40(0.81-2.41)	1.45(0.74-2.84)	1.06(0.47-2.36)	0.99(0.59-1.67)	0.97(0.41-2.31)	0.76(0.39-1.46)
Stayed the same	ref	ref	ref	ref	ref	ref	ref	ref	ref	ref
Increased	1.04(0.67-1.62)	0.97(0.46-2.03)	1.13(0.73-1.73)	1.00(0.62-1.61)	1.20(0.79-1.82)	2.04(1.25-3.31)	1.33(0.74-2.39)	1.08(0.72-1.61)	0.85(0.44-1.65)	1.26(0.80-1.99)
Non-smoker	0.70(0.50-0.99)	0.76(0.43-1.35)	0.79(0.55-1.12)	0.84(0.58-1.23)	0.81(0.58-1.13)	1.19(0.80-1.77)	0.76(0.47-1.25)	0.86(0.63-1.18)	0.67(0.40-1.11)	0.71(0.50-1.03)

Supplementary Table 9. Association between social class and changes in financial situation during the March-July 2020 lockdown (adjusted for ethnicity, age at time of questionnaire, home ownership, maternal and partner education, parity, maternal age and maternal marital status). N=2557

	**NS-SEC social class at age 23 years**			
	**Higher managerial, administrative and professional occupations**	**Intermediate occupations**	**Routine and manual occupations**	**Never worked and long-term unemployed**
	**N=1126**	**N=616**	**N=579**	**N=236**
**Change in employment**				
Employed with the same or more hours	ref	ref	ref	ref
Reduced hours	ref	0.87(0.57-1.34)	1.45(0.96-2.18)	1.54(0.87-2.75)
Furlough/paid or unpaid leave	ref	1.39(1.02-1.91)	2.38(1.73-3.26)	2.10(1.30-3.38)
Stopped working during pandemic	ref	0.98(0.58-1.67)	2.68(1.63-4.42)	2.83(1.45-5.50)
Not working pre-pandemic	ref	1.21(0.72-2.05)	2.11(1.27-3.51)	5.38(3.05-9.49)
**Financial situation**				
Worse off	ref	0.98(0.73-1.31)	1.45(1.08-1.96)	1.45(0.95-2.23)
No change	ref	ref	ref	ref
Better off	ref	0.99(0.76-1.29)	0.81(0.61-1.08)	0.81(0.53-1.23)
**Claimed benefits since pandemic**	ref	0.95(0.65-1.41)	1.45(0.99-2.10)	2.06(1.24-3.43)
**Used rent/mortgage deferral since pandemic**	ref	1.28(0.83-1.97)	1.15(0.72-1.85)	0.73(0.30-1.79)

Supplementary Table 10. Association between Adverse Childhood Experiences score and changes in financial situation during the March-July 2020 lockdown (adjusted for ethnicity, age at time of questionnaire, home ownership, maternal and partner education, parity, maternal age and maternal marital status). N=2707

	**Number of Adverse Childhood Experiences between 0-16 years**			
	**0**	**1**	**2-3**	**4 or more**
	**N=504**	**N=679**	**N=980**	**N=544**
**Change in employment**				
Employed with the same or more hours	ref	ref	ref	ref
Reduced hours	ref	0.80(0.50-1.27)	0.95(0.62-1.45)	0.83(0.49-1.38)
Furlough/paid or unpaid leave	ref	0.96(0.66-1.41)	1.28(0.90-1.81)	1.51(1.02-2.24)
Stopped working during pandemic	ref	1.00(0.57-1.77)	1.28(0.75-2.18)	1.37(0.68-2.78)
Not working pre-pandemic	ref	1.06(0.61-1.84)	1.16(0.68-1.98)	1.66(0.91-3.01)
**Financial situation**				
Worse off	ref	0.98(0.69-1.39)	1.26(0.92-1.72)	1.45(0.98-2.14)
No change	ref	ref	ref	ref
Better off	ref	0.89(0.67-1.19)	0.95(0.71-1.26)	0.93(0.66-1.31)
**Claimed benefits since pandemic**	ref	1.37(0.90-2.08)	1.13(0.75-1.69)	1.32(0.81-2.15)
**Used rent/mortgage deferral since pandemic**	ref	0.72(0.41-1.28)	1.10(0.67-1.82)	1.19(0.64-2.22)

Supplementary Table 11. Association between individual Adverse Childhood Experiences and changes in financial situation during the March-July 2020 lockdown (unadjusted). N=2707

	**Adverse Childhood Experience**									
	**Physical abuse**	**Sexual abuse**	**Emotional abuse**	**Emotional neglect**	**Bullying**	**Violence between parents**	**Parental substance abuse**	**Parental mental illness or suicide**	**Parental criminal conviction**	**Parental separation**
	**N=622**	**N=163**	**N=659**	**N=529**	**N=646**	**N=604**	**N=330**	**N=1183**	**N=280**	**N=780**
**Change in employment**										
Employed with the same or more hours	ref	ref	ref	ref	ref	ref	ref	ref	ref	ref
Reduced hours	1.02(0.70-1.48)	1.27(0.69-2.32)	1.03(0.71-1.50)	1.41(0.98-2.03)	1.15(0.79-1.67)	0.74(0.47-1.15)	1.52(0.95-2.42)	0.87(0.64-1.18)	0.77(0.41-1.46)	1.08(0.75-1.54)
Furlough/ paid or unpaid leave	1.30(0.99-1.72)	1.61(1.02-2.55)	1.29(0.99-1.69)	1.44(1.08-1.92)	1.29(0.99-1.68)	1.41(1.06-1.89)	1.39(0.95-2.04)	1.09(0.87-1.37)	1.20(0.78-1.83)	1.48(1.16-1.90)
Stopped working during pandemic	2.15(1.41-3.27)	0.97(0.41-2.32)	1.13(0.70-1.81)	0.95(0.55-1.63)	1.19(0.79-1.81)	0.84(0.47-1.51)	1.87(1.05-3.33)	1.02(0.70-1.46)	1.01(0.50-2.02)	0.84(0.52-1.37)
Not working pre-pandemic	1.43(0.96-2.13)	1.23(0.61-2.48)	1.19(0.78-1.82)	1.75(1.16-2.66)	1.31(0.90-1.92)	1.27(0.81-1.99)	1.15(0.62-2.13)	1.28(0.92-1.80)	0.85(0.42-1.72)	1.53(1.05-2.23)

**Financial situation**										
Worse off	1.35(1.03-1.77)	1.41(0.88-2.25)	1.05(0.79-1.38)	0.97(0.74-1.28)	1.12(0.86-1.46)	1.04(0.78-1.37)	1.45(1.02-2.08)	1.20(0.96-1.51)	1.32(0.88-1.99)	1.18(0.91-1.52)
No change	ref	ref	ref	ref	ref	ref	ref	ref	ref	ref
Better off	0.91(0.71-1.16)	1.20(0.77-1.85)	1.08(0.84-1.38)	0.81(0.62-1.05)	0.93(0.74-1.18)	0.86(0.67-1.10)	1.14(0.81-1.62)	1.01(0.82-1.24)	1.28(0.86-1.92)	0.89(0.70-1.13)
**Claimed benefits since pandemic**	1.15(0.85-1.56)	1.21(0.70-2.08)	1.09(0.79-1.50)	0.95(0.67-1.35)	1.11(0.80-1.52)	0.93(0.65-1.35)	1.35(0.89-2.05)	0.98(0.75-1.28)	1.10(0.67-1.81)	0.99(0.73-1.33)
**Used rent/mortgage deferral since pandemic**	1.16(0.77-1.75)	1.13(0.52-2.45)	0.90(0.56-1.44)	1.02(0.62-1.70)	1.23(0.83-1.81)	1.47(0.96-2.24)	1.16(0.65-2.08)	1.23(0.86-1.76)	1.23(0.66-2.29)	1.43(0.96-2.13)

Supplementary Table 12. Association between individual Adverse Childhood Experiences and changes in financial situation during the March-July 2020 lockdown (adjusted for ethnicity, age at time of questionnaire, home ownership, maternal and partner education, parity, maternal age and maternal marital status). N=2707

	**Adverse Childhood Experience**									
	**Physical abuse**	**Sexual abuse**	**Emotional abuse**	**Emotional neglect**	**Bullying**	**Violence between parents**	**Parental substance abuse**	**Parental mental illness or suicide**	**Parental criminal conviction**	**Parental separation**
	**N=622**	**N=163**	**N=659**	**N=529**	**N=646**	**N=604**	**N=330**	**N=1183**	**N=280**	**N=780**
**Change in employment**										
Employed with the same or more hours	ref	ref	ref	ref	ref	ref	ref	ref	ref	ref
Reduced hours	0.99(0.67-1.45)	1.12(0.6-2.10)	0.96(0.65-1.42)	1.36(0.93-1.99)	1.11(0.76-1.63)	0.68(0.42-1.09)	1.42(0.84-2.39)	0.81(0.59-1.11)	0.71(0.37-1.37)	0.96(0.65-1.42)
Furlough/ paid or unpaid leave	1.19(0.89-1.60)	1.43(0.89-2.32)	1.21(0.91-1.60)	1.34(0.99-1.80)	1.24(0.94-1.62)	1.25(0.91-1.72)	1.04(0.67-1.60)	0.97(0.77-1.23)	1.01(0.64-1.61)	1.19(0.90-1.58)
Stopped working during pandemic	2.32(1.49-3.60)	0.90(0.36-2.22)	1.08(0.66-1.76)	0.98(0.56-1.73)	1.21(0.79-1.87)	0.82(0.44-1.54)	1.82(0.94-3.50)	1.00(0.69-1.46)	1.03(0.48-2.20)	0.78(0.45-1.33)
Not working	1.28(0.83-1.96)	1.03(0.49-2.17)	1.06(0.68-1.66)	1.67(1.08-2.57)	1.19(0.79-1.80)	1.18(0.72-1.91)	0.87(0.43-1.79)	1.14(0.80-1.62)	0.66(0.30-1.43)	1.29(0.85-1.96)

pre-pandemic										
**Financial situation**										
Worse off	1.35(1.01-1.79)	1.46(0.91-2.36)	1.04(0.78-1.40)	0.98(0.74-1.30)	1.13(0.86-1.48)	1.03(0.77-1.39)	1.39(0.94-2.07)	1.19(0.94-1.49)	1.35(0.88-2.06)	1.18(0.89-1.57)
No change	ref	ref	ref	ref	ref	ref	ref	ref	ref	ref
Better off	0.93(0.72-1.21)	1.27(0.80-2.02)	1.12(0.86-1.45)	0.88(0.66-1.15)	0.96(0.76-1.23)	0.87(0.67-1.14)	1.18(0.80-1.73)	1.06(0.86-1.31)	1.33(0.87-2.03)	0.99(0.76-1.28)
**Claimed benefits since pandemic**	1.11(0.81-1.53)	1.17(0.67-2.05)	1.05(0.75-1.47)	0.94(0.66-1.35)	1.10(0.79-1.53)	0.89(0.61-1.31)	1.32(0.82-2.13)	0.98(0.74-1.29)	1.09(0.64-1.83)	0.97(0.70-1.35)
**Used rent/mortgage deferral since pandemic**	1.18(0.77-1.81)	1.06(0.46-2.43)	0.86(0.54-1.39)	0.94(0.55-1.62)	1.17(0.78-1.76)	1.42(0.90-2.24)	1.04(0.53-2.06)	1.19(0.81-1.74)	1.18(0.60-2.34)	1.28(0.82-2.01)
