# Supplementary material for: Socioeconomic position and adverse childhood experiences as risk factors for health-related behaviour change and employment adversity during the COVID-19 pandemic: insights from a prospective cohort study in the UK
Source: BMC Public Health. 2022 Sep 24;22:1820. doi: 10.1186/s12889-022-14184-8 (PMC9509623; doi:10.1186/s12889-022-14184-8)
Supplement: Supplementary file 2 — Additional file 2. Additional demographic variables. Dichotomous ACE indicators. Auxiliary variables for multiple imputation. [file 12889_2022_14184_MOESM2_ESM.docx]

**Supplementary File**

**Additional methodological details**

**Additional demographic variables**

We included education and income variables in the complete case participant characteristics in Supplementary Table 3. “Monthly take-home pay” refers to the participants self-reported take-home pay each month in English pounds, after the removal of tax and national insurance payments at age 25 years. “Obtained GCSE grades A*-C” refers to a self-reported variable from a questionnaire completed when participants were aged 18 years old. ﻿GCSEs are qualifications obtained at age 16 in the UK, which was the end of compulsory education for this cohort.

**Dichotomous ACE indicators**

Multiple questions fed into each ACE indicator. If an ACE was reported in one or more question, it was assumed to have been experienced, even if there were inconsistencies in responses. ACE indicators were mostly derived from data collected prospectively, with the inclusion of some retrospective self-reported measures, particularly for sexual abuse where the reported prevalence in prospective data is very low.

**Auxiliary variables for multivariate multiple imputation**

Of 110 possible auxiliary variables, the following 24 had at least 50 observations within each level of that variable for both males and females, and so were used in the multiple imputation models (terms in brackets refer to the original ALSPAC variable labels):

- Birthweight (kz030_org)
- Gestation (kz029_org)
- Pre-pregnancy weight and BMI (dw002_org, dw042_org)
- Home ownership status (a006_org)
- Mother’s age at delivery (mz028b_org)
- Parity (b032_org)
- Mother’s marital status (a525_org)
- Mother’s and partner’s highest educational qualifications (c645a_org, c666a_org, pb325a_org, pb342a_org)
- Indicators of mother’s and partner’s mental health conditions (b370_org, c600_org, pb260_org, t3255_org, t5360_org, t5404)
- In last year, father became homeless (fa3322_org)
- Mother or partner smoked, used cannabis/hard drugs or suffered alcoholism (t5412_org, t5510_org, fa5411_org, fa5510_org)
- Young person’s age when partners have used physical force such as pushing, slapping, hitting or holding them down (ypa5005_dup)
